# Identification of nodulation‐related genes in *Medicago truncatula* using genome‐wide association studies and co‐expression networks

**DOI:** 10.1002/pld3.220

**Published:** 2020-05-16

**Authors:** Jean‐Michel Michno, Junqi Liu, Joseph R. Jeffers, Robert M. Stupar, Chad L. Myers

**Affiliations:** ^1^ Bioinformatics and Computational Biology University of Minnesota St. Paul Minnesota; ^2^ Department of Agronomy and Plant Genetics University of Minnesota St. Paul Minnesota; ^3^ Department of Computer Science and Engineering University of Minnesota Minneapolis Minnesota

**Keywords:** bioinformatics, co‐expression, genome‐wide association studies, *Medicago*, nodulation

## Abstract

Genome‐wide association studies (GWAS) have proven to be a valuable approach for identifying genetic intervals associated with phenotypic variation in *Medicago truncatula*. These intervals can vary in size, depending on the historical local recombination. Typically, significant intervals span numerous gene models, limiting the ability to resolve high‐confidence candidate genes underlying the trait of interest. Additional genomic data, including gene co‐expression networks, can be combined with the genetic mapping information to successfully identify candidate genes. Co‐expression network analysis provides information about the functional relationships of each gene through its similarity of expression patterns to other well‐defined clusters of genes. In this study, we integrated data from GWAS and co‐expression networks to pinpoint candidate genes that may be associated with nodule‐related phenotypes in *M. truncatula*. We further investigated a subset of these genes and confirmed that several had existing evidence linking them nodulation, including MEDTR2G101090 (PEN3‐like), a previously validated gene associated with nodule number.

## 1 INTRODUCTION

The ability of legumes to form symbiosis with nitrogen‐fixing rhizobial bacteria makes legumes an integral part of managed and natural ecosystems. A better understanding of the genetic basis of legume–rhizobia N‐fixation may enable researchers to improve nitrogen fixation in these systems, thereby increasing N‐input into agricultural systems. *Medicago truncatula* has served as a model species for understanding the genetic and molecular basis of nodule formation and nitrogen fixation (Young & Udvardi, 2009). These studies have, however, largely relied forward genetic approaches that have used screens that identify de novo mutations of major effect. It is likely that many genes that have minor effects on the number of nodules plants form or the quantity of efficiency of N‐fixation have yet to be identified (Smil, 1999). Genome‐wide association studies (GWAS) provide a means to identify naturally occurring alleles that might be responsible for more minor, or quantitative, variation in the formation and efficiency of the legume–rhizobia N‐fixing symbiosis (Curtin et al., 2017).

Despite the promise of GWAS, the ability of association analyses to identify causative genes is limited by linkage disequilibrium among causative and non‐causative variants as well as statistical false negatives and false positives. The genetic resolution of GWAS is directly linked to the amount of historical recombination that has occurred among the genomes of the individuals that comprise the association panel. Many populations, particularly for species that are not obligately outcrossing, have experienced rapid increases in population size, or have strong population structure; the amount of recombination is limited; and causative variants can be in strong linkage disequilibrium with non‐causative variants. The consequence of this LD is that large genetic intervals can be identified, but GWAS provides no means to identify the specific gene that underlies this variation (Breseghello & Coelho, 2013; Flint‐Garcia et al., 2005; Visscher, Brown, McCarthy, & Yang, 2012). For most traits, the amount of phenotypic variation that can be explained by statistically significant GWAS associations is far less than the genetic variance of the trait (Visscher et al., 2012), which suggests that GWAS suffer from many false negatives that arise due to alleles with small effects not meeting stringent statistical cutoffs (Johnson et al., 2010; Sham & Purcell, 2014; Storey & Tibshirani, 2003). Conversely, lowering the statistical threshold introduces false positives that can lead to mistaken assumptions about the genetic basis of phenotypic variation and hamper downstream functional validation efforts focused on specific candidate genes (Korte & Farlow, 2013).

Except for model species (Lamesch et al., 2012), most genes remain functionally uncharacterized. In fact, even in many model species, the functional characterization is limited to putative function. The lack of functional characterization limits the biological information available to interpret a candidate gene's effect on a specific phenotype. Co‐expression networks provide a powerful resource for understanding gene function; genes that are co‐expressed, that is, have strong edges in co‐expression networks, are likely to have related biological functions. As such, co‐expression modules allow one to link genes of unknown function to genes of known function, on the basis of expression, thereby allowing one to establish a functional context for a gene, even when formal annotations do not exist.

Schaefer et al. (2018) recently described a new framework, Co‐Analysis of Molecular Components, or Camoco. Its goal is to integrate co‐expression networks with GWAS as a means to identify candidate genes associated with phenotypes. In maize, they conducted several GWAS analyses to identify SNPs associated with elemental accumulation in seeds. They further built three distinct co‐expression networks, two from publicly available data and one from root tissue designed to represent the phenotype measured in the respective GWAS. Using GWAS alone, they were able to identify significant markers associated with regions of the genome, but, in most cases, they were left with hundreds of markers per trait that often implicated linked genomic regions that could not be resolved to individual candidate genes. Application of this framework to maize seed expression and phenotypic variation in elemental composition revealed that the Camoco method is better able to identify and prioritize candidate genes associated with elemental accumulation than GWAS alone.

Here, we apply this framework to *M. truncatula,* using publicly available expression datasets, and markers from a previously published GWAS focused on nodulation traits. We demonstrate that the Camoco framework, originally established in maize, indeed generalizes to other species and traits and provides an effective means of pinpointing candidate causal genes associated with nodulation.

## 2 MATERIALS AND METHODS

### 2.1 *Medicago* experimental design and sample extraction

Three accessions from the Medicago HapMap Project (HM56, HM101, and HM340) were grown in greenhouse conditions in a 2:1 mixture of Turface and LP5. Rhizobium strains *Ensifer meliloti* (KH46c) and *E. medicae* (WSM419), as well as nitrogen, were applied to the soil shortly after planting. Tissues were harvested and frozen in liquid nitrogen 31 days after planting. RNA was extracted using the Qiagen RNeasy Plant Mini Kit (Product ID: 74903). Individual nodules were pooled and extracted as a single sample for each plant.

### 2.2 Generation of expression data

RNA from 138 samples was sequenced by the University of Minnesota's Genomic Center using Illumina HiSeq2500 100‐bp single‐end reads. One sample required resequencing (L88), which resulted in 125‐bp reads. Samples were barcoded and multiplexed using Illumina TruSeq HT adapters. Fastq files were checked with Fastqc version 0.11.5, and adapters were trimmed using cutadapt version 1.8.1 with non‐default parameters ‐m 40 and ‐q 30 (Andrews, 2010; Martin, 2011). Reads were then aligned to 4.0 gene models, and reference (http://jcvi.org/medicago/) using STAR 2.5.3a (Dobin et al., 2013), then filtered based on unique mapping scores, sorted and indexed using samtools version 1.6 (Li et al., 2009). FPKM values were generated using Cufflinks version 2.2.1 using non‐default parameters of ‐I 20000 and ‐‐min‐intron length 5. Raw sequencing files are publicly available on the NCBI SRA (PRJNA327225 and PRJNA449544).

### 2.3 Co‐expression network construction and genome‐wide association study integration

**FIGURE 1:**
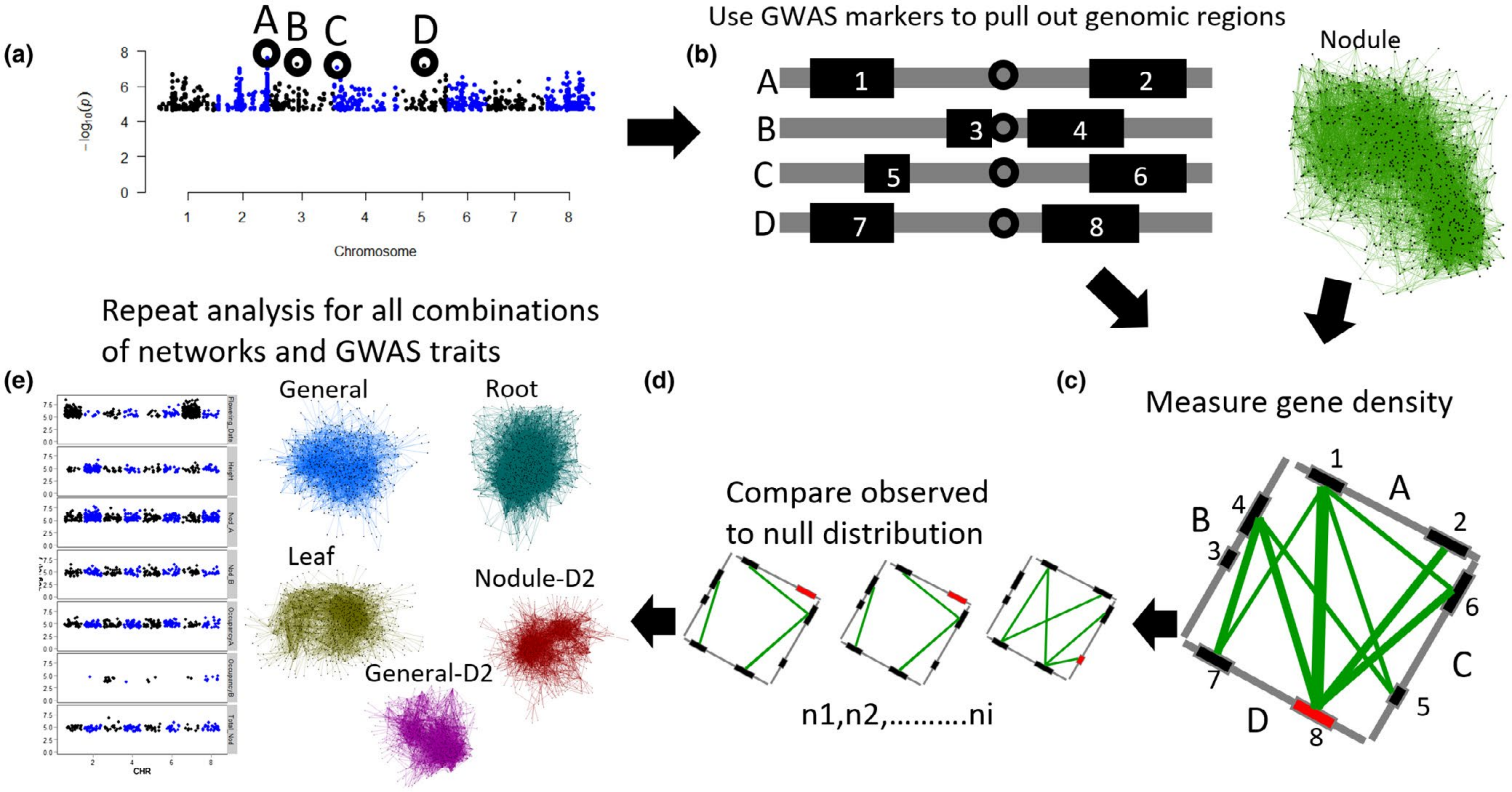
GWAS and co‐expression pipeline using Camoco. (a) Manhattan plot represents DNA markers used as input for Camoco, bold black circles represent a subset of markers used for illustrative purposes. (b) Regions along a chromosome from previously selected markers are represented as gray bars, genes are represented as black rectangles. Black circles represent a SNP from (a). (c) Genes from previously identified intervals are then selected from the co‐expression network for per‐gene network density measurements. Colored lines represent the strength of co‐expression between two genes in a co‐expression network. Wider lines represent gene pairs that are more strongly co‐expressed. The red box represents the current gene being measured for density. (d) Per‐gene density measurement of random subnetworks equal in size to the testing set. (e) Other GWAS traits and networks used for analysis

Methods used were similar to those in the previously mentioned co‐expression GWAS integration study (Schaefer et al., 2018, 2018). Briefly, Camoco takes a set of SNPs as input and uses their location within a genome, as well as the number of genes flanking a marker within a given window size to extract gene lists for testing (Figure 1). If there are multiple significant SNPs appearing within the same window, then all but the most significant SNP are discarded (Table S1). Once genes are selected for testing, each gene is then measured to see how well it is co‐expressed with other genes also linked to the significant markers associated with the trait of interest. Once a network statistic (either density or locality, see Schaefer et al., 2018; Schaefer et al., 2018) is generated, Camoco will resample (1,000 times) a random set of genes equal in size to the test set to establish a null distribution for estimating significance of the observed statistic. To account for the varying amount of linkage disequilibrium across the genome, we used 10, 20, and 50 kb window sizes and 1, 2, and 5 flanking genes (Branca et al., 2011). Any gene that had an FDR < 0.35 was called “candidate” and included in further analysis.

FPKM expression tables were used as input into Camoco (https://github.com/schae234/Camoco) using the 4.1 reference genome. Non‐default parameters used to build each network included rawtype=“RNASEQ,” max_gene_missing_data = 0.5, max_accession_missing_data = 0.5, min_single_sample_expr = 1, min_expr = 0.001, quantile = False, max_val = 300, sep= “,”. Network health statistics were generated using GO terms from (http://jcvi.org/medicago) and 1,000 bootstraps. SNPs were integrated into Camoco using built‐in functions, and per gene, density measurements were run with 1,000 bootstraps. Figures were created using ggplot2 (Wickham, 2006).

## 3 RESULTS AND DISCUSSION

### 3.1 Integration of nodule‐focused genome‐wide association study with co‐expression networks

To identify candidate genes associated with nodulation traits, we used a previously published GWAS (Stanton‐Geddes et al., 2013) and two publicly available RNA‐seq datasets (NCBI SRA accessions PRJNA327225 and PRJNA449544). The GWAS consisted of 226 M*. truncatula* accessions that were grown in replicate and phenotyped for five nodulation traits, and flowering time, trichrome density, and height. By manually inspecting the 50–200 SNPs with the most significant *p*‐values, the authors discovered several genes near significant SNPs that were previously associated with nodulation traits (Stanton‐Geddes et al., 2013). Similar to other GWASs, the authors focused on genes that either contained or were directly adjacent to significant markers even though, in some cases, other genes were also plausible candidates given their linkage to the significant markers (Branca et al., 2011). We selected a subset of the traits from this study, along with markers meeting a specific significance threshold, to serve as input for the GWAS/co‐expression Camoco pipeline (Table S1).

As a basis for our co‐expression networks, we used two publicly available RNA‐seq expression datasets. The data included 138 samples consisting of three genotypes, three tissues, four rhizobium treatments, and presence–absence of nitrogen (Table S2). We built six co‐expression networks using Camoco (https://github.com/LinkageIO/Camoco; Schaefer et al., 2018, 2018). Four of the six networks were constructed from a single tissue type (Leaf, Root, Nodule, Nodule‐D2), and the other two networks (referred to as the “General” network and “General‐D2” network) were constructed from a combination of tissue types (Table S3). The networks labeled “D2” were constructed using data from project number PRJNA327225 within the NCBI SRA, while the other networks were constructed using data from project number PRJNA449544. The diversity of tissue types within each co‐expression network allows for the detection of signals corresponding to different biological processes that may have remained undiscovered if all samples were combined into one large network (Schaefer, Briskine, Springer, & Myers, 2014; Schaefer et al., 2018). The total number of genes that passed the co‐expression network construction phase was relatively consistent among the four networks, with each network consisting of roughly 22,000 genes (Table S3).

To test whether the networks were capturing biologically meaningful relationships, we measured the enrichment in each network for known biological relationships. Using sets of genes annotated to with the same Gene Ontology (GO) term, the relative density (how highly an established set of functionally related genes are co‐expressed with each other) was compared with density values of randomly sampled gene sets of the same size. All six networks demonstrated functional enrichment of at least 10‐fold (Figure S1), indicating that many more GO terms exhibited evidence of co‐expression than expected by chance for all six networks.

Using the six co‐expression networks and selected number of GWAS markers (Table S1), we applied the Camoco pipeline to prioritize candidate causal genes. Briefly, Camoco (Schaefer et al., 2018, 2018) evaluates candidate genes physically linked to significant GWAS markers on the basis of their co‐expression with genes linked to other significant GWAS markers. The assumption driving this analysis is that causal genes are likely to exhibit strong co‐expression relationships with other genes associated with the trait. Camoco is depicted in Figure 1, and the details of this analysis are provided in Methods section. All genes reported by Camoco with an FDR < 0.35 were considered candidate genes and included in further analysis.

**FIGURE 2:**
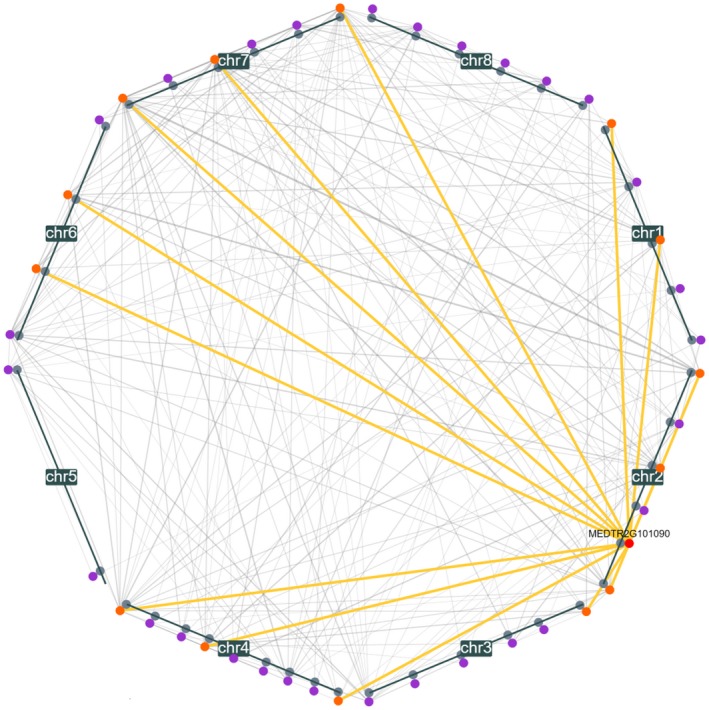
Nodule_A discoverable genes in the Nodule‐D2 network. Chromosome‐centric diagram of the connectivity of discoverable genes (FDR < 0.35), focused on co‐expression neighbors of the candidate, MEDTR2G101090, within the Nodule‐D2 network for the Nod_A trait. Gray circles represent GWAS markers, colored circles represent genes, with MEDTR2G101090 in red, its first neighbors in orange, and other discoverable genes in purple. Gray lines represent co‐expression between genes (minimum *Z*‐Score of 2.5); the wider the line, the stronger the co‐expression between genes. Yellow lines represent the connections of MEDTR2G101090 to its first neighbors

Camoco analysis identified 331 high‐confidence candidate genes across all GWAS trait and network combinations at an FDR < 0.35 (Table S4). Several of these candidate genes were also discovered at more stringent cutoffs (110 at FDR < 0.2, 24 at FDR < 0.1, and 3 genes at FDR < 0.05; Table S4). Analysis of the Nod_A trait (number of nodules within the top 5 cm of roots) with the Nodule‐D2 network revealed a high amount of network connectivity between other high‐confidence candidate genes. To illustrate the basis for highly prioritized candidate genes, we highlight the observed co‐expression relationships for MEDTR2G101090 (Figure 2), which was one of the top prioritized candidate genes for the Nod_A. MEDTR2G101090 is near a significant GWAS marker and is highly co‐expressed with genes linked to significant GWAS markers on several other chromosomes (Figure 2), suggesting that the Camoco framework is discovering meaningful relationships.

### 3.2 Importance of trait and tissue specificity in co‐expression networks

**FIGURE 3:**
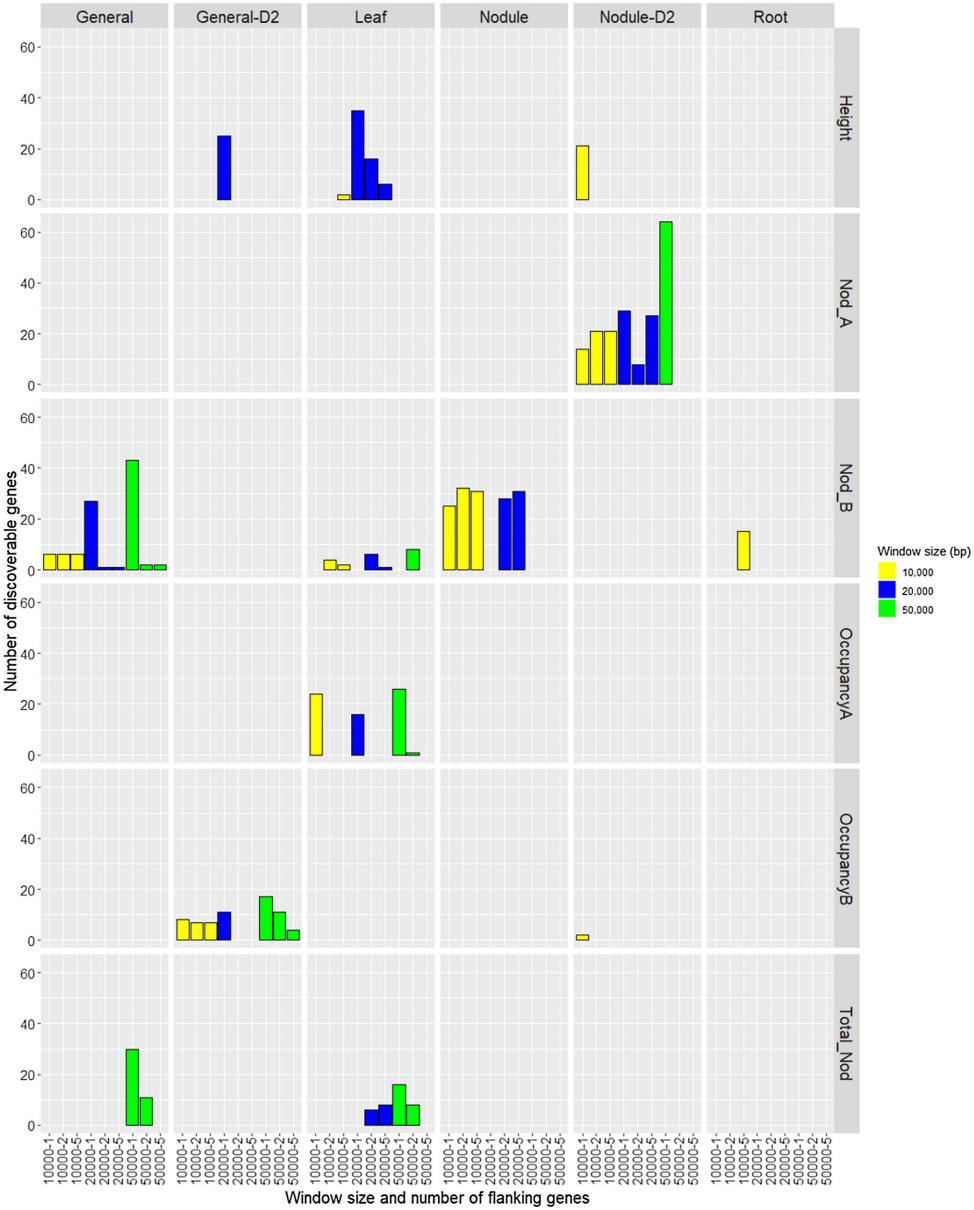
Co‐expression/GWAS discoverable gene summary. Number of discoverable genes (FDR < 0.35) obtained from co‐expression/GWAS integration. Colors represent the window size parameters used for our analysis. Each square in the grid represents a different co‐expression network (horizontal axis) trait (vertical axis) combination

The number of high‐confidence candidate genes discovered by Camoco varied considerably across different combinations of traits, networks, and parameters (Figure 3). Interestingly, the nodule‐based Nodule‐D2 co‐expression network combined with the Nod_A trait yielded the most high‐confidence candidate genes across all network–trait combinations, which likely reflects a strong match between the tissue in which expression was measured and the phenotype of interest (in this case, both focused on nodules). Surprisingly, the root‐based network performed the worst even though we expected strong biological relevance for nodulation‐based traits. This result could be due to the time point at which RNA was extracted from the roots. For example, if RNA was extracted at an earlier time point when nodules were still early in development, there may have been more informative expression patterns, allowing for the discovery of candidate genes.

The leaf network was the only network that consistently identified candidates for the height trait. While this is biologically unsurprising, this network also discovered significant genes for a few nodulation traits, suggesting that there are processes detectable in leaf tissue with relevance to nodulation. The General network, which consisted of the largest number of samples and tissue types, only generated a few candidates for the Nod_B phenotype.

These results suggest that the context from which the co‐expression network was derived, and its relation to the GWAS phenotype, play an important role in determining whether the Camoco approach is able to prioritize high‐confidence genes. Notably, our results suggest that combining many different types of tissue into one large network does not perform as well as a smaller, more concise, tissue‐focused networks, even though it is based on a larger set of expression profiles. One reason for this is that combining expression data from very different contexts introduces more variation across each gene's profile, but that variation likely reveals generic modules that represent large sets of genes that simply are expressed in the same subsets of tissues. In contrast, networks derived from specific tissues capture subtler covariation that reflects co‐regulated genes functioning in processes relevant to that tissue that may otherwise be lost in larger sets of expression profiles.

### 3.3 High‐confidence candidate genes are not always close or the most significantly associated marker

A common approach to interpreting GWASs is to manually inspect the most significant markers and look for candidates that are closest in proximity to the marker of interest. Unfortunately, the closest genes to GWAS markers may not always be the ones that are causally driving the association with the phenotype. When looking at the height trait in the leaf network, we see an increase in signal (i.e., number of Camoco‐identified high‐confidence candidate genes) as we increase the number of flanking genes surrounding each marker (Figure S2). When the window size is increased from 10 kb to 20 kb, we see that the signal drastically increases, indicating that there are genes further out from the marker that are highly co‐expressed with a subset of these genes. However, when an even larger 50‐kb window is used, no high‐confidence genes are reported. The loss of signal at the largest interval (50 kb) is expected as the number of potential candidate genes per locus increases sharply (the large majority of them being false positive as one considers candidates further from the locus peak). Ultimately, this large number of false candidate genes obscures the identification of co‐expression relationships among true causal genes, and the approach no longer works. This analysis suggests that several of the GWAS loci implicated for these traits are likely driven by causal genes that are not directly adjacent to the GWAS peaks.

Similarly, the constraint of only focusing on the most significant markers (e.g., derived from extremely conservative significance cutoffs on the association test) leaves other candidates that are truly associated with the phenotype neglected. The Camoco framework can provide filtering of false positives at lower significance thresholds, by integrating information from the co‐expression network. For instance, if we used the commonly assumed stringent GWAS *p*‐value cutoff of 5 × 10^–8^ (Fadista, Manning, Florez, & Groop, 2016), this would result in two GWAS markers from the Nod_A phenotype, which does not provide enough context for an approach like Camoco to prioritize candidate genes. Instead, we applied a less conservative threshold (*p*‐value < 3 × 10^–5^), which resulted in 292 SNPs, which was able to produce several high‐confidence candidate causal genes, which would have otherwise been ignored (Table S1). In general, of course, the number of markers produced at any confidence threshold will depend on the trait's genetic architecture and the study design, but this analysis suggests that the Camoco approach can better produce candidate genes with less conservative thresholds on marker association.

### 3.4 Identification of nodulation‐related genes using co‐expression and GWAS

**FIGURE 4:**
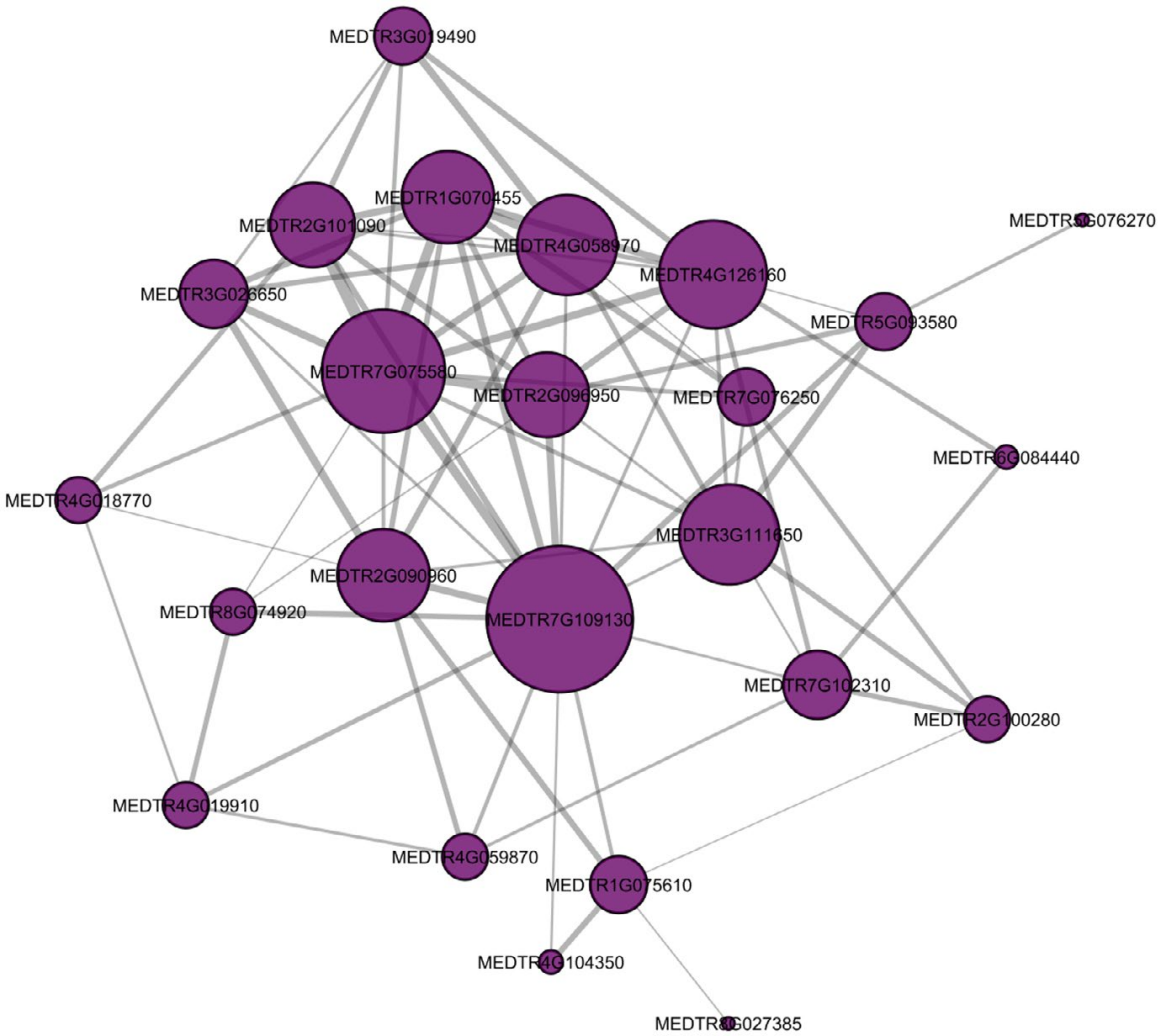
Overlap of Nod_A candidates in the Nodule‐D2 network. Candidate genes for the Nodule‐D2 network for the Nod_A trait. Purple circles represent genes, and gray lines represent co‐expression between genes (minimum *Z*‐Score of 2.5). The larger the circle, the more connections it has with other genes. The wider the line, the stronger the co‐expression between genes

To identify a small set of the most promising high‐confidence candidate genes for more investigation, we further narrowed candidate genes lists for the Nod_A trait by focusing on genes that were consistently discovered across different parameter settings. Using the Nodule‐D2 network, we narrowed the candidate gene lists by limiting candidate genes to those that appeared in at least three out of the nine combinations of parameter settings (10, 20, 50 kb genome window size by 1, 2, 5 flanking genes); this process resulted in 25 genes for further investigation (Table 1). When viewing the strength of co‐expression between these 25 genes within the nodule network, it was observed that the majority of the genes were connected and formed a single module (Figure 4).

**TABLE 1:** List of genes that were discoverable across all six parameters (10, 20, and 50 kb and 1, 2, and 5 flanking genes) for the Nod_A phenotype using the Nodule‐D2 network

Gene	Number of connections (*Z*‐score 2.5 or higher)	SNP_position	GWAS ‐log10(*p*.val)	Rank (out of 292)	Annotation
MEDTR2G101090	8	chr2:43448968	7.591607	1	Drug resistance transporter‐like ABC domain protein
MEDTR8G074920	4	chr8:31665171	6.753532	11	Receptor‐like kinase theseus protein
MEDTR2G100280	4	chr2:43061039	6.743592	12	RNA exonuclease‐like protein
MEDTR4G018770	4	chr4:5776217	6.509395	19	GDP‐mannose transporter GONST3
MEDTR3G026650	6	chr3:8183997	6.177657	53	GDP‐fucose protein O‐fucosyltransferase
MEDTR4G059870	4	chr4:22091245	5.827601	114	C2H2 and C2HC zinc finger protein, putative
MEDTR4G019910	4	chr4:6362962	5.7494	139	SnoaL‐like domain protein
MEDTR5G076270	1	chr5:32504251	5.707181	156	Auxin response factor 2
MEDTR6G084440	2	chr6:31605458	5.678609	161	DUF1666 family protein
MEDTR2G090960	9	chr2:39088095	5.657328	171	TCP family transcription factor
MEDTR4G104350	2	chr4:43099392	5.512627	210	DNA polymerase III subunit gamma/tau
MEDTR7G102310	6	chr7:41285876	5.493289	220	Rhodanese/cell cycle control phosphatase superfamily protein
MEDTR5G093580	5	chr5:40860194	5.415629	252	Co‐factor for nitrate, reductase and xanthine dehydrogenase
MEDTR3G019490	5	chr3:5482913	5.410043	257	S‐locus lectin kinase family protein
MEDTR7G109130	16	chr7:44591633	5.381151	270	P‐loop nucleoside triphosphate hydrolase superfamily protein
MEDTR8G027385	1	chr8:9668134	5.239786	350	Endomembrane Family Protein
MEDTR4G126160	11	chr4:52449376	5.231223	358	Cytokinin oxidase/dehydrogenase‐like protein
MEDTR7G076250	5	chr7:28686036	5.221541	366	Zinc finger, C3HC4 type (RING finger) protein
MEDTR4G058970	10	chr4:21744831	5.102555	448	Homeodomain leucine zipper protein
MEDTR7G075580	13	chr7:28296141	5.067043	470	Cytochrome P450 family protein
MEDTR1G075610	5	chr1:33462984	5.06158	474	Cyclin‐dependent kinase
MEDTR2G096950	8	chr2:41430755	5.050944	485	Kinase 1B
MEDTR1G070455	9	chr1:31235133	5.044264	491	WRKY transcription factor
MEDTR3G111650	10	chr3:52196531	5.019337	507	Hypothetical protein
MEDTR1G080690	0	chr1:35874811	5.009149	517	TPX2 (targeting protein for Xklp2) family protein

Interestingly, among those 25 candidate genes from the Nod_A analysis was PEN3‐like (MEDTR2G101090; Table 1), a gene that was associated with the most significant GWAS marker for the Nod_A trait (Stanton‐Geddes et al., 2013). Functional validation of PEN3‐like using CRISPR and Tnt1‐mutated plants previously confirmed that loss of function of this gene resulted in decreased nodule number (Curtin et al., 2017). Another strong candidate among these 25 within the module was the hub gene (gene with the highest number of connections), MEDTR7G109130, which is annotated as a P‐loop nucleoside triphosphate hydrolase superfamily protein whos gene family is known to play a role in nodulation (Jayaraman, Richards, Westphall, Coon, & Ané, 2017). Notably, the marker linked to this gene was ranked 270 out of 292 significant markers for this trait, so it may not have been prioritized for further analysis without using the Camoco approach.

Because multiple co‐expression networks were able to support the discovery of strong candidate genes for the Nod_B trait, we defined a short list of high‐confidence candidates for this trait by requiring high‐confidence genes to be consistently prioritized as high‐confidence candidates across all networks for the Nod_B trait and discovered across 4 or more parameter settings (Table 2). One promising gene, MEDTR1G012530, appeared as a candidate for 9 out of the 20 parameter settings that resulted in at least one candidate gene discovery. This gene is annotated as a TPX2 (targeting protein for Xklp2) family protein and has been shown to be highly expressed during nodule formation (Jardinaud et al., 2016). Another promising candidate, MEDTR4G073400, which also appeared as candidate 9 times, is annotated as Synaptotagmins‐1‐related, which play a role in the formation of root nodules (Gavrin, Kulikova, Bisseling, & Fedorova, 2017).

**TABLE 2:** List of genes that were discoverable for at least five different parameters across all networks for the Nod_B trait

Gene	Number of hits across parameters and terms	Annotation
MEDTR4G027195	10	N/A
MEDTR4G035980	10	Pectinesterase/pectinesterase inhibitor
MEDTR1G012530	9	TPX2 (targeting protein for Xklp2) family protein
MEDTR4G073400	9	Synaptotagmin‐1‐related
MEDTR2G073540	8	Cysteine‐rich RLK (receptor‐like kinase) protein
MEDTR1G028960	6	Glycolipid transfer protein (GLTP) family protein
MEDTR1G037520	5	N/A
MEDTR1G040105	5	Methylenetetrahydrofolate reductase
MEDTR2G048855	5	Pentatricopeptide (PPR) repeat protein
MEDTR2G090960	5	TCP family transcription factor
MEDTR2G450720	5	SAM domain (sterile alpha motif) protein, putative
MEDTR3G088820	5	PPR containing plant‐like protein
MEDTR4G087510	5	O‐acetylserine (thiol) lyase
MEDTR5G053950	5	Allene oxide cyclase
MEDTR5G065080	5	PURINE permease
MEDTR5G094290	5	Tubulin‐folding cofactor A
MEDTR6G023600	5	SHORT‐chain dehydrogenase/reductase
MEDTR6G048290	5	PPPDE thiol peptidase family protein, putative
MEDTR7G039370	5	Origin recognition complex subunit 6
MEDTR8G432620	5	Methyltransferase

Overall, these results demonstrate that the integration of co‐expression networks to interpret GWAS results was able to effectively prioritize genes causally associated with nodulation processes. The genes that are directly connected to PEN3‐like would serve as valuable candidates for follow‐up studies due to their similarity in expression profiles across tissues.

## 4 CONCLUSIONS

Using an *M. truncatula* GWAS focused on nodulation traits and expression data from different tissues, rhizobium strains, nitrogen treatments, and accessions, we were able to identify a subset of genes surrounding GWAS markers that are highly co‐expressed with one another. From these lists, we discovered a previously validated nodulation gene PEN3‐like and several other genes whose annotations are associated with nodulation. Uncharacterized genes within our high‐confidence lists are worthy of more in‐depth follow‐up studies using Tnt1 or CRISPR knockouts.

Schaefer et al. (2018, 2018) developed the Camoco framework and integrated co‐expression networks and GWAS in maize in order to capture variation associated with elemental uptake in seeds. Our current study used a higher‐density GWAS that focused on a different phenotype, different plant species, and an expression dataset that was not explicitly created for this study. One common theme between the studies is that the choice of the co‐expression network matters; specifically, tissue‐relevant networks derived from expression variation across diverse genotypes appear to perform the best in ranking candidate genes. This was true in maize, and we report here that this is also true in Medicago. We believe this result is likely to generalize to many other contexts, and it suggests as a community, more emphasis in the generation genotype‐focused networks would be worthwhile if we hope to build resources for functional interpretation of phenotype‐associated variants. It is also important to mention that we were able to generate a panel of high‐confidence candidate genes using two independent datasets that were not generated specifically for this study.

The majority of candidate genes discovered in this analysis would have mostly likely been neglected by traditional GWAS analyses unless they were under the most significant markers. By combining co‐expression networks with GWAS, the functional relationship between genes related to the GWAS phenotype are more likely to be discovered. It is also important to note that based on our analysis, in many cases, the nearest gene to a marker was not the gene predicted to be causally associated with the phenotype.

In general, we demonstrate that the Camoco framework for integrating co‐expression networks with GWAS generalizes beyond the species for which it was originally developed and applied (maize ionomic traits), as it shows utility for prioritizing genes related to nodulation in *Medicago truncatula*. Based on these results, we expect that the approach will generalize to a wide variety of other species and traits as well.

## AUTHOR CONTRIBUTIONS

JMM and CLM designed the experiment. JMM and JRJ performed the bioinformatics. JQL extracted RNA. JMM and CLM wrote the manuscript. All authors read and approved the final manuscript.

## Supporting information

Figure S1

Figure S2

Table S1–S4

Supplementary Material
