# Supplementary figures and images for: Identification of nodulation‐related genes in Medicago truncatula using genome‐wide association studies and co‐expression networks
Source: Plant Direct. 2020 May 16;4(5):e00220. doi: 10.1002/pld3.220 (PMC7229696; doi:10.1002/pld3.220)

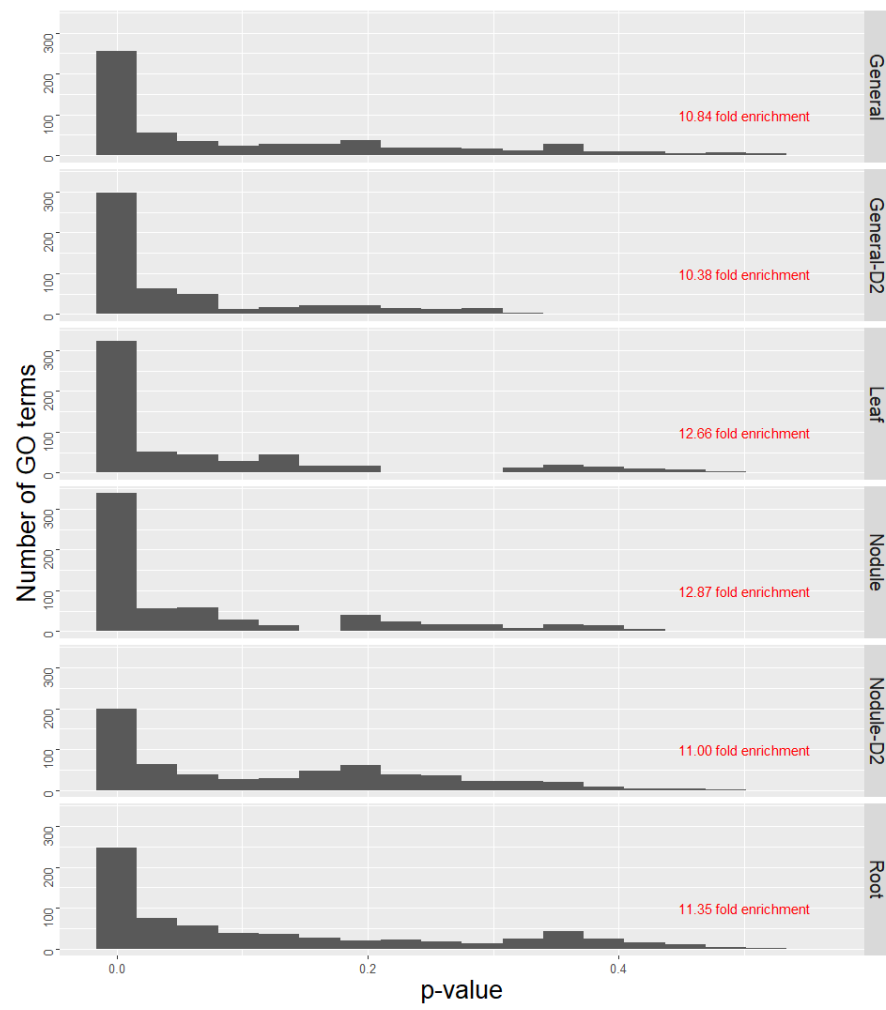

Supplement: Supplementary file 1 — Figure S1 [file PLD3-4-e00220-s001.pdf]
