# Supplementary material for: Identification of nodulation‐related genes in Medicago truncatula using genome‐wide association studies and co‐expression networks
Source: Plant Direct. 2020 May 16;4(5):e00220. doi: 10.1002/pld3.220 (PMC7229696; doi:10.1002/pld3.220)
Supplement: Supplementary file 2 — Figure S2 [file PLD3-4-e00220-s002.pdf]

A)

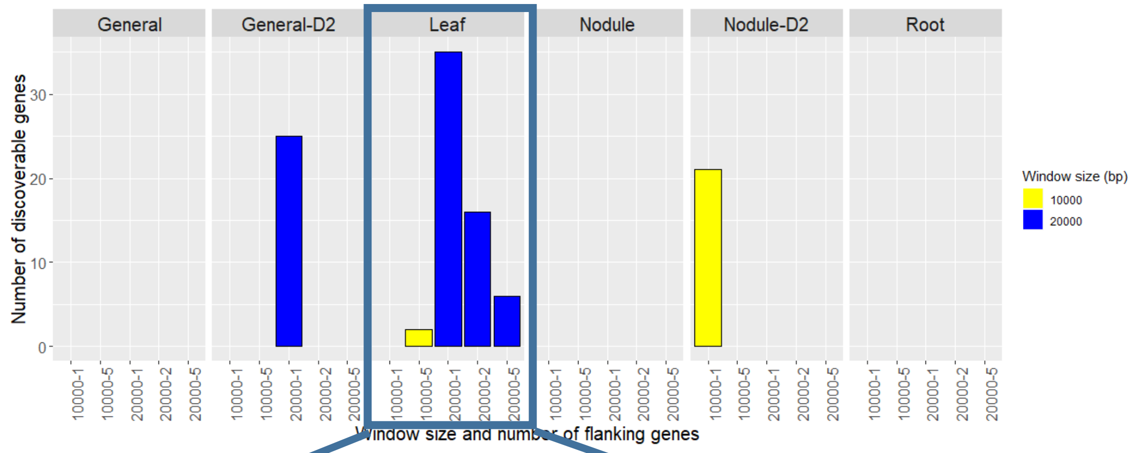

B)

Leaf/Height GWAS									
**Window size (bp)**	**10000**			**20000**			**50000**		
**Number of flanking genes**	**1**	**2**	**5**	**1**	**2**	**5**	**1**	**2**	**5**
**Starting number of SNP's**	197			197			197		
**SNPs after collapse**	139			133			127		
**Number of genes tested**	138	182	198	165	257	326	196	363	663
**Number of discoverable genes**	0	0	2	35	16	6	0	0	0
