# Supplementary material for: Identification of nodulation‐related genes in Medicago truncatula using genome‐wide association studies and co‐expression networks
Source: Plant Direct. 2020 May 16;4(5):e00220. doi: 10.1002/pld3.220 (PMC7229696; doi:10.1002/pld3.220)
Supplement: Supplementary file 4 — Supplementary Material [file PLD3-4-e00220-s004.pdf]

Reviewer #1:

This manuscript is an extension study of the original "Camoco" paper. But it applied Camoco on Medicago, using co-expression networks derived from different tissues to assess its performance on detecting candidate genes involved in nodulation-related traits. Some potential speculations from results could be interesting, such as "if RNA was extracted at an earlier timepoint when nodules were still early in development, there may have been more informative expression patterns, allowing for the discovery of candidate genes". Testing performance of Camoco using coexpression networks derived from different tissues and sequential thresholds on agronomic traits would be informative for users. The manuscript is overall good and useful for people to understand tissue-wide and trait-wide application of Camoco, but authors missed citations for supporting their points in some places. Some statements should be strengthened, such as the first paragraph in the results section. I have pointed out as below.

1. "For all but a few model species, most genes remain functionally uncharacterized...". There are actually literatures talking about this point in some species. Why not cite those papers? Such as "The Arabidopsis Information Resource (TAIR): improved gene annotation and new tools" ... please try to add citations for supporting your point.

Arabidopsis and Maize are considered model species, a lot of genomes are being sequenced to this day when most annotations remain uncharacterized. I will take out the “few” to create more of a separation

2. Authors missed details in "By manually inspecting the 50200 SNPs with the most significant p-values, the authors discovered several genes near significant SNPs that were previously associated with nodulation traits", how many SNPs in total? Instead of "several genes", what is the actual number of genes you identified for each trait?

I think this was just a misinterpretation of the text. The text reads“50-200 most significant SNPs”, but the hyphen was at the end of the line, so we understand how that might have been confusing

3. "3 at FDR < 0.05: 3 genes ..." , "3 genes" seems duplicated in here

We thank the reviewer for catching this—this has been corrected.

4. X tick labels for figure 3 are shifted, please relabel them.

Thanks for noting this—we have fixed it.

5. Please unify the format of number like 1000 to 1,000 or make this consistent.

We have updated the text to use commas in all such numbers.

6. "...commonly assumed stringent GWAS p-value cutoff of ...", you need citations to support this.

We agree and have added appropriate citations.

7. Table 1, you mentioned six parameters, but you explained five parameters in the caption, please make this consistent.

Thanks for noting this—this error has been corrected.

8. Figure 2, what does the yellow line indicate?
The yellow line is the lines connecting to the first neighbors. We added this information to the legend.

Reviewer #2:

Using established Camoco, this article extensively examines the relationship between co-expression networks and GWAS studies to discover candidate genes. Some minor changes are suggested below.

1. In figure 1A, although the circled markers were used for illustrative purpose, point B and point D seem to be false positive as they are the only significant points (no clustering pattern)

The extent of the clustering near a true positive genetic association depends on the degree of linkage disequilibrium around that locus, which can vary dramatically across a genome. Thus, it is difficult for one to conclude that point B and D are likely false positives without more context. Also, as the reviewer points out, this is simply an illustrative example.

2. Did you do any filtering based on gene expression before constructing the co-expression network? Most co-expression networks are based on the calculation of correlations among genes which could be biased by expression levels of genes.

As mentioned in the manuscript, we filtered genes that did not meet minimum expression level criteria across the set of lines we considered. These parameters are mentioned in the "Co-expression network construction and genome-wide association study integration" section and are consistent with our previous study (Schaefer et al.).

3. Why did you pick up FDR cutoff as 0.35?

Our approach reports a false discovery rate for each candidate gene, and this information is included in the supplemental files we included with the manuscript. Readers can choose to apply whatever FDR they wish in interpreting the resulting gene lists. In the text, we report the number of discoveries made at several different cutoffs, Results section: "Camoco analysis identified 331 high-confidence candidate genes across all GWAS trait and network combinations at an FDR < 0.35 (Table S4). Several of these candidate genes were also discovered at more stringent cutoffs (110 at FDR < 0.2,

24 at FDR < 0.1 and 3 genes at FDR < 0.05: 3 genes).” We think our reporting the results across a range of thresholds, as well as including the FDR estimates of all individual candidates is a reasonable approach.

4. For those connected genes identified in this article, do they share similar functions? (GO, pathway, or transcription factor)

The genes we discuss in the main text of the manuscript are related to functions related to the nodulation life cycle. We did perform a GO enrichment analysis on this set of genes as well, but didn’t find strong enrichment. There are potentially a number of different explanations for this, but this may reflect the fact that the Medicago genome is still relatively poorly annotated (one of the key motivations for developing our approach).

Reviewer #3:

The manuscript "Identification of nodulation-related genes in M. truncatula using GWAS and co-expression networks" integrated the expression correlation of genes located within a user-defined flanking region to empower the statistic power in finding candidate genes. The idea was adapted from a previous developed pipeline 'Camoco' published in Plant Cell by the same authors in corn. Nonetheless, this manuscript is the first assessment for soybean and for nodulation-related signals. Although the scientific integrity and exploration outcome may help the GWAS community or the soybean community in future research using this strategy, some suggestions are listed as following:

The authors estimated the co-expression networks of 1, 2, or 5 flanking genes with an interval (10Kb, 20Kb, or 50Kb) of a significant SNP (a co-expression network within a tissue? or a co-expression network among tissues?) and the significant SNPs into Camoco for prioritizing candidate genes. The authors seemed to be reluctant to re-described the details such as the definition of density and locality, and referred many places in the manuscript back to the previous publication in the Plant Cell. Re-describe the pipeline or details by a different person can provide a unique understanding to Camoco, and it will be very helpful for readers to get familiar with this tool by two different and distinct publication. Otherwise, the future citations may eventually attribute back to Schaefer et al. 2018 and the publication in the Plant Direct will become negligible. As the manuscript appeared to be shorter than many other articles in the Plant Direct, the authors may consider to re-introduce or review the methodology of Camoco from a different aspect.

We appreciate the reviewer’s suggestion about re-introducing Camoco here, but we prefer to refer readers back to the original manuscript for details of the method. The main purpose of this paper is to demonstrate that the method produces interesting candidate genes for a different species beyond the setting for which it was originally developed. We think this is a significant result worth highlighting on its own.

Moreover, GWAS in soybean system has been well established and other than using dummy variables for window size, it is recommended that the authors may implement the linkage

disequilibrium of each chromosome (Vuong et al. 2015 BMC Genomics) instead of 10, 20, or 50Kb. Improvement in using all genes or near genes within the LD block may provide advanced understanding and novelty rather than using the conventional setting of intervals as well as 1, 2, or 5 genes.

We agree with the reviewer that it would be ideal to use data on the linkage disequilibrium at each locus rather than using fixed window sizes/flanking gene limits, and this is something we plan to address in the future. However, this is beyond the scope of the current manuscript, where we intended to demonstrate that the Camoco method, as previously published, produces strong candidate genes.

Secondly, the authors used FPKM values as input for Camoco, but it is unclear were the FPKM values directly from the previous publication or were re-analyzed by the authors? No details in the Materials and Methods. FPKM values were based on pair-end sequencing, and how many of the public expression data were pair-end? Did the previous publication used the same mapping software and criterions? Is it necessary for the authors to re-analyzed and synchronize the quantification parameters, and maybe also applied better normalization statistics such as TPM before entering Camoco?

The details of the FPKM quantification and parameters were provided in the methods section "Generation of expression data". Table S2 also provides barcodes for paired end sequencing. The data was also made publicly available on the SRA, which is cited in our manuscript, so more information can be found there.

Thirdly, the authors identified 331 "high-confidence" candidate genes at FDR < 0.35, meaning the expectation of making type I error (expectation of a false discovery or incorrect rejection on null hypothesis) was 35%, most 4 out of 10 candidate genes will be wrong. The setting at 0.35 therefore may be too high, and the rationale should be justified especially when the authors also mentioned that there are 24 candidate genes at FDR 0.1. Can the MEDTR2G101090 or other experiment-proved nodulation-related gene be found at FDR 0.1 or even lower FDR?

As discussed in the response to Reviewer #2 above, our approach reports a false discovery rate for each candidate gene, and this information is included in the supplemental files we included with the manuscript. Readers can choose to apply whatever FDR they wish in interpreting the resulting gene lists. In the text, we report the number of discoveries made at several different cutoffs, Results section: "Camoco analysis identified 331 high-confidence candidate genes across all GWAS trait and network combinations at an FDR < 0.35 (Table S4). Several of these candidate genes were also discovered at more stringent cutoffs (110 at FDR < 0.2, 24 at FDR < 0.1 and 3 genes at FDR < 0.05: 3 genes)." We think our reporting the results across a range of thresholds, as well as including the FDR estimates of all individual candidates, is a reasonable approach. The tolerable level of FDR depends on the reader and what the reader intends to do with the results. In our experience, there are many settings where a list generated with a criteria of FDR < 50% would be sufficient given the fact that one is going to combine the resulting candidates with prior knowledge or other data before investing in further experiments. Regarding the specific candidate gene

MEDTR2G101090, this gene was discovered with an FDR ranging from 0.15 to 0.33 across different parameter combinations.

In addition, the language and description can be more careful to remove inaccurate or redundant information. For example, in the conclusion paragraph, the authors stated that" ...more emphasis in the generation genotype-focused networks would be worthwhile..." but the study did not assess differences between soybean genotypes. The sentence should be revised as " ...more emphasis in the generation"of tissue-specific" networks would be worthwhile..." as the authors mentioned in one of the subheading. Another example is that the scientific name of Medicago on the title should be spelled out. Due to no line number presented in the current version, minor editorial suggestions are not listed in this review.

We thank the reviewer for providing these editorial suggestions. We're confused by the comment that our "study did not assess difference between soybean genotypes". Expression profiles evaluated here were composed of data from three different Medicago genotypes, which were important for candidate gene discovery.

Table 1. mentioned six parameters but the parentheses included only five?

Thanks for pointing this out—we've fixed this issue.

Table 2. mentioned five parameters with no details. Are these the 10Kb, 20Kb, 1, 2, or 5 genes?

We have clarified this as requested.

The rationale of choosing five parameters as mentioned in Table title is confusing... If the setting of 1 gene nearby the significant SNP with 10Kb, then other settings will be filtered out regardless the parameters at 2 or 5 genes in 20 KB? In other words, 1 gene in 10Kb of a significant SNP is a subset of 5 genes in 20Kb. Clarification is needed.

Thanks for the suggestion—this has been fixed.

The SNP dots in the Manhattan plot below -log10 of 4 disappeared in Figure 1a and 1e. Why?

The Camoco method first thresholds the GWAS marker association list at a user-specified threshold. For this example, we used a cutoff of $p < 1 \times 10^{-4}$. For illustrative purposes, the later panels focus on subnetworks involving only 4 of the SNPs meeting this threshold (more is difficult to show). In the actual Camoco run, all SNPs above the significance threshold would be evaluated for increased co-expression density/locality.

Reviewer #4:

Michno et al. present an analysis using co-expression networks to prioritize candidate genes identified by proximity to significant SNPs from a published GWAS study. The method, Camoco, was recently published in a separate paper. While the original paper was benchmarked using

maize data, this current study was partly an experiment to see whether the Camoco approach can be extended to other species (specifically, Medicago truncatula) and experimental setups.

I think the paper was well-written and easy to read. The methods were sound and the conclusions are supported by the results. I think that the Camoco approach is a nice way to help narrow down a list of candidate genes, with some caveats. In this regard, the authors do a good job of presenting examples of when it works well and when it doesn't, offering explanations of what the issues might be and potential solutions. They discuss how to choose RNA-seq datasets to make it more likely to detect robust signals and different parameters to tweak such as window size and number of flanking genes around significant GWAS SNPs. I think the recommendations in this paper should be valuable information for researchers who intend to adopt Camoco or similar co-expression approaches for narrowing down candidate genes.

I only have one minor request. In the text, the two SRA project IDs that the RNA-seq samples belong to are listed. However, in Table S2 it is not clear which samples came from which of the two projects. One could go on the SRA and make the inference, but it would be more convenient to just have the information in the table.

The sample names in column A shows the different nomenclatures and SRA repositories can be browsed. We have also added an extra column specifying the NCBI SRA project number for each respective sample.

Reviewer #5:

1. In the manuscript, it states that two publicly available RNA-seq datasets were used (second paragraph of results section) but there is no study cited. Yet in the methods section there is a description of tissue harvest, RNA extraction and sequencing. The text is very misleading. Please expand on the experimental design in the results section to clarify.

The study used to generate the data never ended up in a published manuscript. We reached out to the generators of the data and included the details of the experimental methods in the in the methods section. The same information can also be found on the NCBI SRA project page where we uploaded the same information.

2. Is bias in cis expression for candidate genes accounted for in the analysis? In the original Camoco paper, genes found co-expressed with candidate genes that were located nearby were removed. This may be a non-optional parameter in the Camoco pipeline but it's worth mentioning here as well since it is likely not a well-known process.

Yes, this is the default operation of Camoco.

3. As you are applying Camoco to a new species from the original manuscript, it would be very helpful to include the full code used to run Camoco including the RNA-seq processing pipeline. This would be useful for anyone wanting to apply this method to their data. This can be easily done through a Github repository for example.

Since this is an extension of Camoco but in a different species, we decided to include all of the parameters used to run the software from FPKM generation (Generation of

expression data section), to co-expression network construction (Co-expression network construction and genome-wide association study integration section). We didn't need to create any custom functions/code to run this pipeline, which is why we don't cite a separate Github repository.

Minor Comments

1. Figure 1A – This panel might look better if the maker density was extended down the y-axis and a significance line included.
   The significance of Figure 1A is meant to illustrate that we are selecting a subset of markers. While the reviewer does suggest a nice alternative, we do not think that change will enhance the interpretation of selecting a subset of SNPs.
2. 2. Figure 1B – In the legend, please include a note that the black circle represents the location of the SNP from panel A.

   We appreciate the feedback and have modified the figure accordingly.

3. Figure 1 – Please avoid red and green in order to make the figure color blind friendly.

   Since the red and green areas are highlighting different items (density vs. a gene), we do not believe this to be an issue. The differentiator would be between black and red genes which would be colorblind friendly.

4. Figure 2 – This figure is difficult to see, the font size is very small and it is hard to see the difference in line width. Also, there is no mention in the legend about the yellow lines?
   The yellow lines connect the first neighbors. We added this information to the legend.

Grammar INTRO - 'largely relied ON forward genetic' - 'number of nodules PLANTS form or the quantity OR efficiency'

RESULTS - 'sets of genes annotated to __ the same Gene Ontology' - 'which also appears as A candidate 9 times' - 'prioritize genes causally associated with THE nodulation processes'

CONCLUSIONS - 'more emphasis in the generation OF genotype-focused' - 'would have MOST likely been neglected'

We thank the review for grammatical corrections--we have modified the manuscript.
